# Optimizing Microwave‐Convectional Drying of Probiotic‐Infused Apple Snacks: Impact on Quality Attributes and Predictive Modeling with Equations and Artificial Neural Network

**DOI:** 10.1111/1750-3841.70348

**Published:** 2025-06-24

**Authors:** Derya Dursun Saydam

**Affiliations:** ^1^ Department of Nutrition and Dietetics İstanbul Yeni Yüzyıl University İstanbul Turkey

**Keywords:** microwave‐convectional drying, neural network modeling, osmotic dehydration, probiotic‐infused apple snack, texture attributes

## Abstract

Fruit drying in the modern food industry requires easily operable, energy‐saving, inexpensive, and efficient drying technologies. Furthermore, these technologies are critical for snacks that meet the various dietary sensitivities and requirements of consumers and provide a benefit to overall well‐being. In this context, the aim was addressed to produce snacks of apple infused with probiotic microorganism *Lactobacillus rhamnosus* GG. Fresh osmotically dehydrated apple cubes with ultrasound were dried by convectional and microwave‐convectional technologies in line with an experimental plan with varying power and temperature levels. The impacts of the treatments on the color and texture quality attributes of the dried apple cubes and the survival of probiotic bacteria were investigated. The Pearson and principal correlation analysis between color and texture parameters showed that hardness (HA) and color changes were proportional to each other. Alongside the microwave‐assisted hot air drying, notable alterations were detected particularly in the products’ gumminess, chewiness, HA properties, and redness and yellowness values. In regard to statistical analysis of six mathematical equations used to model the kinetic data, the Midilli and others model offered the best fit for the drying operations. Feed‐forward neural network approach was employed to describe the association between drying process inputs and outputs, and it consistently showed its predictive capacity with a high *R* ^2^ value of 0.96008. For the commercial production of the highest probiotic‐grade dried apple cubes with quality characteristics, 90 W microwave energy at 50°C may serve as a successful drying operation.

**Abbreviations**
ANN: Artificial Neural Network
CH: chewiness
CO: cohesiveness
GU: gumminess
HA: hardness
MR: moisture content ratio
PC1: Principal Component 1
PC2: Principal Component 2
PCA: principal component analysis
RE: resilience
RMSE: root mean square error
SEC: specific energy consumption
SP: springiness
US‐OD: ultrasound assisted osmotic dehydration

## 1 Introduction

Apples are a prevalent fruit that can be easily and widely grown all over the world and are rich in vitamins, minerals, dietary fiber, and prebiotics that may contribute to human health. In addition to being very suitable for obtaining familiar products such as juice, gel, vinegar, and jam in the food industry, the texture of the flesh and skin of the apple makes it a very good role model for tailoring innovative products in food research. Probiotic‐enhanced apple products, as functional foods, can be beneficial for digestive health, reducing oxidative stress and improving immune system (Erdoğan and Demirci 2014; Demirci et al. 2017; Bustos et al. 2023). In recent years, consumers interested in gut and general well‐being without formulated products, with various food sensitivities, special health conditions, or in search of new products have a fascination with such products. Manufacturers can satisfy demanding consumers by formulating these products in snack form for practical use. Optimizing drying technologies and kinetics for these snacks to maximize the viability of probiotic cultures, maintain the quality of the finished product, and improve the efficiency of the processes is important as consumer demand for functional foods continues to grow (Li et al. 2018; Nuñez et al. 2024).

In the ever‐evolving food industry, particularly in snack production sector, numerous drying technologies are employed to improve the quality and shelf life of products. Of these technologies, hot air and microwave drying stand out as two methods that have their own set of drawbacks when used individually, but when combined, they transform these challenges into advantages and reinforce one another (Yoğurtçu 2014). Hot air drying can efficiently process large quantities of food, which is relatively simple in technology, cost‐effective, and appropriate for a variety of foods, including perishables like fruits and vegetables (Calín‐Sánchez et al. 2020; Rosdan et al. 2024). However, the method uses high temperatures and takes a long time to dry, which can adversely affect the color and texture of snacks that are key quality attributes for snacks affecting consumers’ initial impressions. In contrast, microwave drying technology using microwave energy significantly reduces drying time and ensures a more uniform drying pattern. As a result, it can preserve both the nutritional content and quality parameters of snack products more effectively than convectional hot air methods. However, this technology can present challenges, such as higher initial investment costs and the need for precise control over process parameters to avoid overheating or uneven drying. It is critical to combine the strengths of the two technologies, hot air drying for cost‐effectiveness and productivity and microwave drying for enhanced snack quality features, depending on product requirements and manufacturers’ objectives (Yoğurtçu 2014).

Hybrid drying methods are combined with pretreatments to help drying technologies achieve the intended results. When used in conjunction as pretreatments, ultrasound and osmotic dehydration can yield noteworthy outcomes. The ultrasound‐assisted osmotic dehydration (US‐OD) process aims to reduce the amount of water in the fruits and thus preserve the basic quality parameters and the viability of probiotic microorganisms as much as possible by subjecting the fruits to drying at lower temperature levels and for shorter time. The existence of such studies is mentioned in the literature by Nowacka et al. (2012), Goula et al. (2017), Pantelidou et al. (2021), Ahmad et al. (2023), and Ferreira et al. (2024). Furthermore, this application, which has the effect of improving and accelerating subsequent processes (dehydration procedures, drying methods, extraction, etc.), has already been examined in numerous studies in food field and has been developed (Ricce et al. 2016).

Moreover, the kinetics of drying plays a significant role in the overall success of the production process. The drying rate affects how quickly moisture is removed from the apple and consequently influences the texture and color attraction of the final product. The application of advanced drying technologies such as microwave‐assisted drying has shown promising results. These methods not only reduce the drying time significantly but also enable better control over temperature, minimizing heat damage to sensitive probiotic microorganisms. Studies have demonstrated that optimizing drying kinetics can result in higher retention of both quality parameters and the viability of probiotic microorganisms, which are critical for consumer acceptance and health benefits (Alibaş 2007; Horuz et al. 2017; Calín‐Sánchez et al. 2020). Optimization is mostly carried out mathematically. Valuable and meaningful drying models are good at doing this task. However, deeper and more meaningful perspectives can be captured with different approaches. For these purposes, artificial intelligence studies can provide an innovative and developmental impact on food research (Huang et al. 2007).

To sum up, the production of probiotic‐infused apple snacks can be significantly enhanced through the optimal selection of drying technologies and an understanding of drying kinetics. By adopting advanced techniques and focusing on the preservation of the viability of probiotic microorganisms, manufacturers can efficiently produce high‐quality snacks that cater to the growing health‐conscious consumer market that is a growing and promising segment in the food industry. With this overview in consideration, this research aims to:

- investigate the effects of convective and microwave‐convective treatments on color characteristics and various texture attributes that have not been studied and/or compared of apple cubes in the literature,
- analyze and model the drying kinetics using mathematical and neural network approaches,
- determine process efficiency and energy consumption,
- optimize the drying conditions that ensure high viability of probiotic microorganisms, good quality, and moderate energy usage.

## 2 Materials and Methods

### 2.1 Apple Preparation

Apple fruit (*Malus domestica starking delicious*) was purchased from a local market (Gaziantep, Turkey). It was cleaned with water and then peeled and cut into 1 cm^3^ cubes with a kitchen cutting device. To prevent the oxidation, the cubes were prepared right before they were immersed in sucrose solution for US‐OD process.

### 2.2 US‐OD Process

The osmotic dehydration system was set up considering sucrose solution concentration (50%, w/v) and solid (the apple cubes, 50 ± 1.0 g in total) and liquid (the sucrose:water solution) ratio (1:4, w/v). When the osmotic system was prepared, the ultrasonication was started and performed for 10 min in an ultrasonic bath (Intersonic, Min 4 Model, Transducer Pzt = 4, 350 W, 350 W, Turkey) with constant temperature (30°C) and frequency (25 kHz). As soon as the ultrasonication was finished, the osmotic dehydration was continued in a rotary shaker (Innova 40R New Brunswick Scientific, USA) at 180 rpm and 30°C to fill up a total US‐OD time of 3 h (Dursun Saydam et al. 2019).

### 2.3 Impregnation Procedure of Microorganism

Man, Rogosa, and Sharpe (Merck, Germany) was used in liquid and agar forms as a selective medium for *Lactobacillus* species to activate and propagate the probiotic microorganism *Lactobacillus rhamnosus* GG (ATCC 53103). Minor modifications were made to the protocols described by Rodrigues et al. (2018) for cell transfer and inoculum. The inoculations of the bacterium were made with fresh culture (cultivated for 24 h at 30°C) to obtain a minimum viable cell concentration of 10^9^ CFU/mL. Impregnation of the fresh culture into the cubes was carried out for 10 min at 180 rpm, 30°C in the rotary shaker (Innova 40R 156 New Brunswick Scientific, USA). Following each process, viable cell counting was carried out for 48 h at 30°C (Halkman 2005).

### 2.4 Drying Process

A drying plan (Table 1) was developed on the basis of the results of the preliminary drying studies with apple cubes, which identified the microwave power and temperature values that provided the minimum number of probiotic bacteria (10^6^ CFU/g) needed to ensure that the probiotic quality of the finished product could be achieved. Following the impregnation, a hybrid dryer (Arçelik KMF 833 I, Turkey) consisting of microwave (maximum 900 microwave power (W)) and hot air (from 40°C to 280°C) was used for drying treatments of the apple cubes at three temperature degrees (40°C, 50°C, and 60°C) and two microwave output powers (90 and 120 W). During the drying treatments (Table 1), the weight reduction was measured every 20 min using a precision balance (Radwag, PS3500/C/1, Radom, Poland), and energy consumption was measured using a watt meter (Rev, Type 2580, Mömbris, Germany). The drying experiments were continued until the difference in the weight reduction of the apple cubes between the last two data was less than 1%. The dried apple cubes were analyzed for moisture content in wet basis at 105°C for 5 h (AOAC 2023) using a convection oven (JSOF‐100/110 21n‐36‐2013, Korea), viable cell, color parameters, and texture profiles.

**TABLE 1:** Drying plan for the apple cubes.

Experiments	Temperature (°C)	Microwave power (W)
1	40	0
2	40	90
3	40	120
4	50	0
5	50	90
6	50	120
7	60	0
8	60	90
9	60	120

### 2.5 Color Measurement

The color of the apple cubes was measured using a HunterLab Color Flex A60‐1010‐615 (HunterLab., Reston, VA, USA) according to the CIELAB colorimetric system. The parameters of L* (=100, white), a* (>0, redness), and b* (>0, yellowness) were determined at four different points. In addition, the total color change (∆*E*) between fresh and dried apple cubes was calculated using the following formula. The values of fresh cubes are represented by L*_0_, a*_0_, and b*_0_, whereas the values of dried cubes are represented by L*, a*, and b*:

(1) $$\Delta E=\sqrt{\left(\;{\left(L{*}_{0}-L*\right)}^{2}+{\left(a{*}_{0}-a*\right)}^{2}+{\left(b{*}_{0}-b*\right)}^{2}\right)}\;$$

### 2.6 Texture Profile Analysis

Texture profile analysis was performed using a TA.XTplus Tissue Analyzer (Stable Micro Systems, Surrey, UK). The dried apple cubes were measured for hardness (HA), elasticity (springiness, SP), cohesiveness (CO), gumminess (GU), chewiness (CH), and resilience (RE) by double compression with a Dia Aluminium Radiused AACC (P/36R) probe of 36 mm in diameter with a contact surface of 1017.0 mm^2^. The operating conditions were a pretest speed of 1 mm/s, a test speed of 1 mm/s and a posttest speed of 2 mm/s. A force of 20 g was applied for 5 s in “strain” mode to contact 60% of the sample.

### 2.7 Calculation of Moisture Content Ratio

Equation (2) was used to calculate the moisture content ratio (MR) based on the moisture content measurements taken during each 20 min period. In the equation, *M* stands for moisture content at any given time, *M*_0_ for the product's initial moisture content, and *M_e_* for the product's moisture content at equilibrium (Azeez et al. 2017):

(2) $$\mathrm{MR}=\frac{\left(M-M_{e}\right)}{\left(M_{0}-M_{e}\right)}\;$$

### 2.8 Calculation of Drying Rate

The drying rate (g water/g dry solid × m) describing water removed from dry material during drying process period was calculated to determine the rates of the drying curve. Equation (3) expressed that *M_(t_*_+Δ_ *_t_*_)_ is the moisture content in dry material at any time (g water/g dry material), ∆*t* is drying time range, and *t* is drying time (min) (Mishra et al. 2021):

(3) $$\mathrm{Drying}\;\mathrm{rate}=\frac{\left(M_{\left(t+\Delta t\right)}-M_{t}\right)}{\Delta_{t}}\;$$

### 2.9 Calculation of Effective Mass Diffusivity, Biot Number, and Specific Energy Consumption (SEC)

The effective mass diffusivity coefficient of the apple cubes products during the drying period was estimated by using Fick's second law of diffusion (*D*_eff_‐Fick) (Ju et al. 2016). Fick's second law was applied to experimental data to find *D*_eff_‐Fick. The following assumptions were made including the following: (1) Initial moisture content is distributed uniformly throughout the mass of sample, (2) sample is a homogenous cube, (3) shrinkage is negligible, and diffusion coefficient is constant, (4) moisture movement is one dimensional, and (5) external resistance is negligible. The following equation was used to determine *D*_eff_‐Fick (Crank 1979; Meerasri and Sothornvit 2022):

(4)

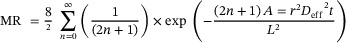

where *n* is the number of terms of the series, *D*_eff_ is the effective diffusivity coefficient (m^2^/s), *t* is drying time, and *L* is the average value of the side length of apple cube. As the drying of apple cubes takes a long time (*n *= 0), Equation (4) can be simplified to Equation (5) using only the first term of the series:

(5)

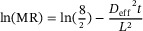




The *D*_eff_‐Fick was determined from the slope of ln(MR) versus drying time (*t*) curve using the following equations:

(6) $$\mathrm{Slope}=\frac{{D_{\mathrm{eff}}}^{2}}{L^{2}}\;$$

The Biot number was determined using the following equations developed by Dhurve et al. (2021):

(7) $$B_{i}=\frac{24.848}{D_{i}}\;$$

(8) $$D_{i}=\frac{v}{k.L}\;$$

where *B_i_* is the Biot number, indicating resistance to moisture diffusion within the samples; *D_i_* is the Dincer number, presenting the relationship between air velocity and the coefficients of heat treatment systems, *v* is air velocity (0.5 m/s), *L* is the thickness of the apple cube (m), and *k* is drying constant (1/s) determined from the best fitted model.

The SEC (kWh/kg) which is the energy utilized per unit mass of moisture removed during the drying process was calculated in the following equation (Torki‐Harchegani et al. 2016):

(9) $$\mathrm{SEC}=\frac{E_{t}}{M_{w}}\;$$

where *E_t_* is the data measured by a watt meter, and *M_w_* is the initial weight of fresh sample (kg).

### 2.10 Modeling Approaches for Kinetic Data

Due to the nonlinear nature of drying kinetic processes, it is very important to select the appropriate modeling approach that can help to understand, predict, and optimize dynamic systems. Accordingly, mathematical equations and Artificial Neural Networks (ANNs) were applied for modeling kinetic data in this study.

#### 2.10.1 Modeling with Mathematical Equations

Six drying curve models were selected and performed for each experiment based on MR values. The equations of the models are given as follows: Page (Sarsavadia et al. 1999), Newton (Azizpour et al. 2014), Wang and Singh (Wang and Singh 1978), Logarithmic (Franco et al. 2017), Midilli and others (Midilli et al. 2002), and Henderson–Pabis (Henderson and Pabis 1961). The models were fitted to the experimental data by SigmaPlot Version 11 software (Erkrath, Germany):

- **Page equation**: $\mathrm{MR}=\exp\;(-kt^{n}$)
- **Newton**: MR=exp(−kt)
- **Wang and Singh**: $\mathrm{MR}=1+at+bt^{2}$
- **Logarithmic**:MR=aexp(−kt)+c
- **Midilli and others**: MR=aexp(−kt)+bt
- **Henderson–Pabis**: MR=aexp(−kt)

#### 2.10.2 Modeling with ANN

For predicting results in nonlinear systems across a range of parameter combinations, the ANN has been regarded as an excellent prediction tool. ANN was used to model the drying process of apple cubes and predict the MR using MATLAB software (version 2017a). The *ANN backpropagation method* was employed by using a *feed‐forward neural network* and the *Levenberg–Marquardt algorithm* for nonlinear activation functions. The data from the drying experiment were divided into three sets: 60% for training, 20% for validation, and 20% for testing. The neural network model for dehydration kinetics was configured with 1 (input layer)–4 (hidden layer)–1 (output layer) architecture. The input and output layers utilized drying time as inputs and MR as the output variable, respectively. These datasets yielded with the highest mean square error and *R* ^2^ (coefficient of determination) values among the various training, validation, and testing datasets used to run the ANN kinetic models. The performance of the ANN model was evaluated by comparing the experimental data with the estimated data using root mean square error (RMSE) and *R* ^2^ values.

**FIGURE 1:**
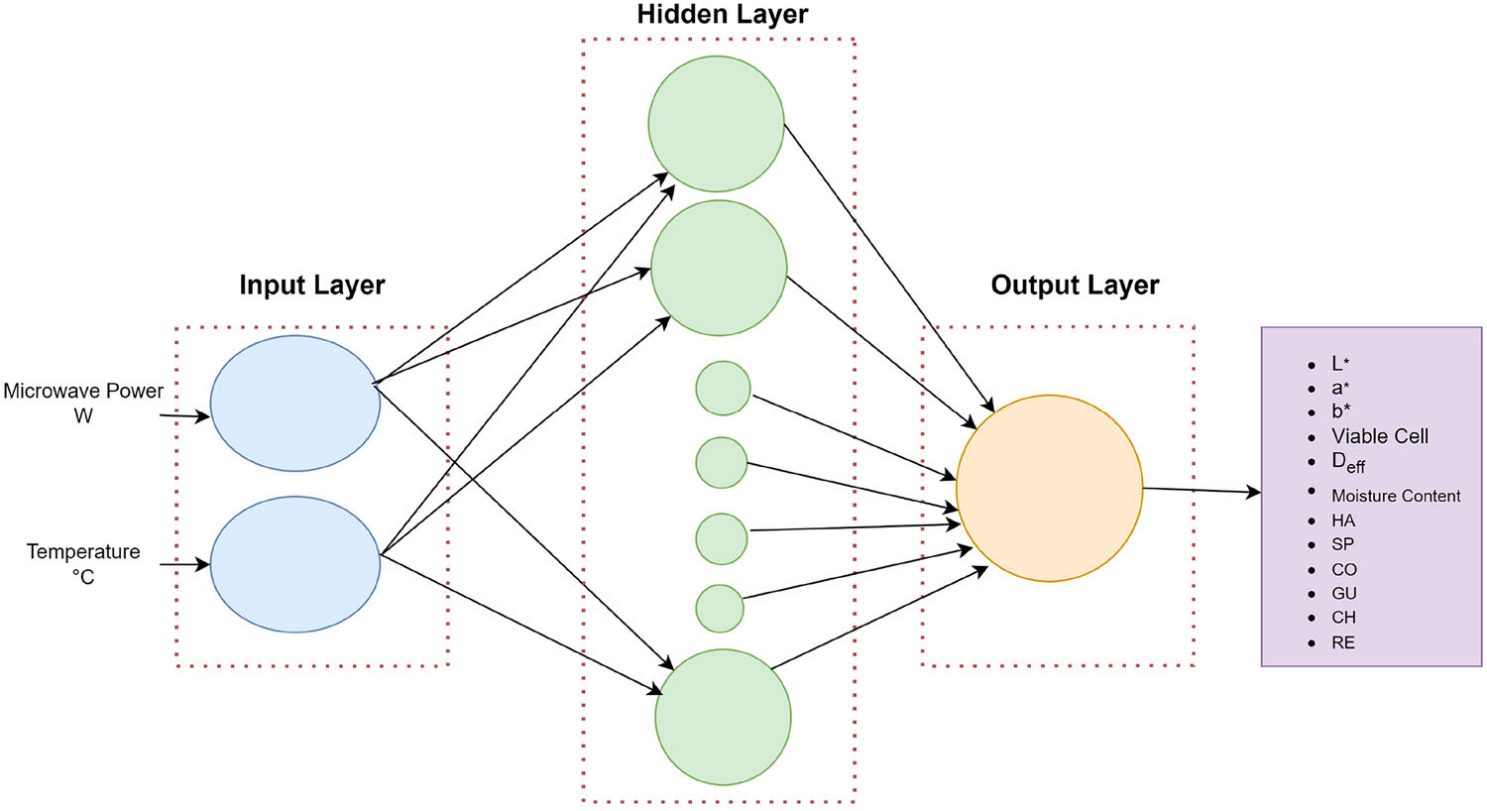
Architecture of the neural network. CH, chewiness; CO, cohesiveness; GU, gumminess; HA, hardness; RE, resilience; SP, springiness.

The graphical user interface of MATLAB loaded the input/output variable datasets into the neural network toolbox. The network was trained using the *Trainlm function*. This training function adjusts the weight and bias values according to the selected algorithm, with the *Levenberg–Marquardt approach* being the most suitable for the data. The connection type for the ANN was a multilayer *feed‐forward backpropagation* learning technique. The adaptation learning function utilized was *Learngdm*. A neuron's input signal was converted to the output signal using the *logsig function*, as shown in the neural network topology in Figure 1. To avoid overfitting, training was terminated when certain conditions were met, including a performance gradient of 10^−7^, six validation checks, and a maximum of 1000 epochs. The datasets used for analyzing the ANNs were categorized into three groups: training, testing, and validation. A total of 70% of the datasets were utilized for training, whereas 15% were allocated for testing and the remaining 15% for validation to evaluate the network's performance. The neural network toolbox in MATLAB software (version 2017a) was employed for the investigation. The input parameters included microwave power (W) and temperature (°C), whereas the output variables included color (L*, a*, b*), texture (HA, SP, CO, GU, CH, RE), effective diffusion coefficient (*D*_eff_), viable cell count, and moisture content. The number of the hidden layers was 10. This learning function employs gradient descent and incorporates bias and momentum weights.

### 2.11 Statistical Analysis

Using the SPSS software, Duncan^a^ and one‐way ANOVA tests were performed for parallel measurements of the analytical analysis at a significance level of 5% (Version 29.0 IBM SPSS Software, USA).

Statistical evaluation of the predicted data for goodness of fitting was performed by RMSE in Equation 10 and *R* ^2^ (obtained from the software). The goodness of fit of the experimental and predicted data was revealed by high *R* ^2^ and by low RMSE values:

(10) $$\mathrm{RMSE}=\sqrt{\frac{1}{N}\sum_{i=1}^{N}{\left(MR_{\mathrm{pre},i}-MR_{\exp,i}\right)}^{2}}\;$$

where the definitions are *N*, number of data; MR_pre_ *_,i_*, predicted model value; MR_exp_ *_,i_*, experimental value.

Principal component analysis and Pearson correlation analysis were performed to be able to relate and understand the interactions between the dependent variables of color and texture parameters using Origin 2018 software (OriginLab, USA). The heat map visualization was generated on the basis of Pearson correlation coefficients.

## 3 Results and Discussion

### 3.1 Effects of Pre‐Drying and Probiotic Bacteria Infusion

To understand the accelerating effect of US‐OD as a pre‐drying process on apple cubes, moisture content change was investigated. There were statistically significant differences (*p* < 0.05) between the results in terms of measured moisture content values. Treatment with US‐OD resulted in an average moisture content decrease of 11.48%, which shows the advantage of US‐OD application. This result is also supported by the study of Jiang et al. (2021) that they showed moisture content decrease via US‐OD as a pre‐drying step of strawberries. On the other hand, an average of 8.61% water absorption from the probiotic microorganism solution occurred in 10 min impregnation process. It can be concluded that in this instance, the US‐OD treatment has a limited impact on the amount of energy consumed and the time taken during the drying process. Santos et al. (2024) reported that a 30 min US treatment at 25°C accelerated the drying process in avocado slices by removing water, thus reducing the drying time. They also performed probiotic microorganism impregnation in a solution at 25°C for 30 min, but no comments on water regain after impregnation were found.

Upon drying at 60°C, the number of viable cells did not meet the quality of being probiotic‐grade (≤10^4^ CFU/g) and was excluded from further experiments. Significant results in moisture content reduction were obtained when the levels of temperature and microwave power in the dryer increased (Table 2). However, it is understood that microwave power became more effective as the temperature of the dryer increased. The process of drying at [50°C + 120 W] provided maximum water removal with moisture content result of 24%. This study supports the phenomena that exposure to microwave application and temperature increases modify products’ dielectric characteristics, which in turn increases the removal of water (Liu et al. 2013). In addition, when food is dried with microwave energy, its inside temperature is higher than its surface temperature, and water transfers more dynamically than when it is dried by convection (Yoğurtçu 2014).

**TABLE 2:** The results of drying procedures and color measurements.

Apple samples/Processes	Duration of processes (*t*, min)	Moisture content (%)	Viable cell (CFU/g)	L*	a*	b*	∆*E*	HA (g)	SP	CO	GU (g)	CH (g)	RE
Fresh apple cubes	*t*_0_	84.20± 0.05^e^	—	74.26± 1.24^e^	3.25± 0.18^b^	27.69± 0.24^c^	3.76	—	—	—	—	—	—
Ultrasonication	*t*_10_	82.37± 0.40^e^	—	74.23± 1.46^e^	1.91± 0.37^a^	24.18± 0.82^a^	7.76	—	—	—	—	—	—
US‐OD	*t*_180_	73.28± 0.81^d^	—	68.20± 0.20^d^	7.24± 0.03^c^	30.44± 0.11^d^	10.19	—	—	—	—	—	—
Drying at 40°C	*t*_440_	40.71± 1.75^c^	2.28 × 10^7^	65.28± 0.65^bc^	9.73± 0.32^ef^	35.12± 0.53^f^	13.34	1049.97± 20.27^a^	0.70± 0.02^ab^	0.78± 0^c^	931.16± 98.35^a^	595.71± 28.42^ab^	0.52± 0^c^
Drying at 40°C + 90 W	*t*_400_	32.44± 8.08^b^	0.51 × 10^7^	66.30± 1.22^c^	10.75± 0.47^g^	37.94± 1.17^h^	14.98	1666.24± 149.55^c^	0.67± 0.01^ab^	0.62± 0.01^a^	1028.56± 109.91^ab^	695.74± 88.96^b^	0.30± 0.01^a^
Drying at 40°C + 120 W	*t*_320_	31.45± 4.08^b^	0.30 × 10^5^	64.53± 0.20^ab^	9.80± 0.18^f^	35.84± 0.12^fg^	14.28	1151.08± 129.25^ab^	0.72± 0.05^b^	0.74± 0.07^b^	661.38± 176.36^a^	712.52± 86.83^b^	0.46± 0.08^bc^
Fresh apple cubes	*t*_0_	86.79± 0.25^e^	—	73.35± 0.45^e^	3.05± 0.11^b^	28.07± 0.20^c^	1.55	—	—	—	—	—	—
Ultrasonication	*t*_10_	82.71± 1.49^e^	—	73.10± 0.11^e^	3.14± 0.09^b^	26.54± 0.36^b^	11.65	—	—	—	—	—	—
US‐OD	*t*_180_	74.75± 1.04^d^	—	64.06± 0.72^ab^	8.71± 0.18^d^	32.25± 0.24^e^	12.05	—	—	—	—	—	—
Drying at 50°C	*t*_360_	35.58± 1.97^bc^	1.20 × 10^6^	64.17± 0.44^ab^	9.12± 0.48^d^	36.51± 0.86^g^	13.87	900.70± 69.39^a^	0.63± 0.02^a^	0.69± 0.03^ab^	621.06± 69.81^a^	392.78± 57.52^a^	0.39± 0.04^abc^
Drying at 50°C + 90 W	*t*_300_	30.45± 5.05^ab^	0.80 × 10^6^	65.19± 0.56^bc^	9.23± 0.19^de^	36.26± 0.14^fg^	13.11	1612.28± 233.55^bc^	0.81± 0.01^c^	0.67± 0^ab^	1410.30± 171.52^b^	1140.86± 126.60^c^	0.31± 0^a^
Drying at 50°C + 120 W	*t*_280_	24.04± 0.64^a^	0.10 × 10^5^	62.99± 0.49^a^	10.68± 0.29^g^	35.62± 0.71^fg^	14.92	1540.99± 53.63^bc^	0.71± 0.01^ab^	0.65± 0.01^ab^	1005.04± 23.56^ab^	716.25± 8.69^b^	0.35± 0.01^ab^

*Note*: a–g: The difference between the means of the values in the same column is at *p* < 0.05. ±: standard deviation.

Abbreviations: CH, chewiness; CO, cohesiveness; GU, gumminess; HA, hardness; RE, resilience; SP, springiness; US‐OD, ultrasound‐assisted osmotic dehydration.

When the drying processes were analyzed in terms of the time to reach a constant weight, a reduction of 80 min was observed when the temperature increased from 40°C to 50°C. It is obvious that the increase in temperature shortens the drying time. When the microwave accompanied the convectional drying, the total drying time decreased at both temperature levels as shown in Table 2. Fan et al. (2018) stated in their review study that microwave application provides a more effective drying due to the fact that the heat penetrates directly into the food and distributes the heat evenly on the food surface and inside. As a result, microwaving reduces the total drying time. Arslan and Özcan (2010), Özkan Karabacak et al. (2015), Ambros et al. (2018), Zura‐Bravo et al. (2019), Kipcak and Doymaz (2020), Tepe and Tepe (2020), and Kidoń and Grabowska (2021) demonstrated similar responses in their studies.

In both drying temperatures applied, it is seen that the drying time decreases as the power increases. Yet, the results showed that the microwave had a greater impact on the total drying time reduction at 40°C operations than it did at 50°C processes. In the light of the studies of Durance and Yaghmaee (2011), Mana et al. (2012), and Ambros et al. (2018), convectional evaporation in slow mode at lower temperature, combined with the effective role of the microwave in dissipating heat, may have resulted in a more controlled and successful sublimation in the removal of water from the apple cubes, thus accelerating drying.

Table 2 shows that the viability of probiotic bacteria is impacted by all of these changes in drying temperature and duration. González et al. (2021) reported similar changes and effects in their studies. Drying at 40°C has a limited effect on the viability of probiotic bacteria. It is observed that the viability rate decreases with the introduction of microwave power. However, this decrease does not make a significant difference compared to the drying process alone at 40°C. It can be considered that microwaves are less effective on the death of bacteria, or their effect is limited when combined with a certain temperature. A greater loss of viability is observed in the drying process with 120 W microwave power. This suggests that microwaves have an increased capacity to kill bacteria. Bacterial viability is significantly reduced when drying at 50°C as opposed to 40°C. This implies that bacteria die out more readily at higher temperatures. At 50°C, the impact of microwave power is also noticeable. The addition of microwave power accelerates the rate at which bacterial viability declines, even while the effect on bacteria increases with temperature. The value here indicates the lowest bacterial viability. Bacterial viability is severely impacted by drying at both high temperatures (50°C) and high microwave powers (120 W). This represents the situation where the combination of temperature and power has the highest bacterial killing capacity.

### 3.2 Color and Texture Profiles of the Dried Apple Cubes

ANOVA results showed that the differences between the means of the color parameters measured in each procedure were significant at *p* < 0.05 (Table 2). During the drying processes, temperature and microwave power had little effect on any of the color parameters, although there was a noticeable increase in a* and b* parameters and a decrease in the L* parameter with the onset of the osmotic dehydration process. Stated differently, microwave and temperature employments had minimal impact on the apple cubes’ redness and lowered brightness, whereas the osmotic dehydration caused significant changes. Jiang et al. (2021), who encountered similar results with the US‐OD process applied to strawberries, explain this situation with the changes and losses of various bioactive pigments and nutrients during the US‐OD process. Further, the application of ultrasound causes new structural formations in the product due to changes in the micro‐channels of the products and the diffusion of external substances such as sugars during the osmotic process, resulting in a decrease in the internal resistance of the products during drying, which may cause color changes (Ricce et al. 2016; Önal et al. 2021). To give apple cubes a consistent and attractive appearance in the eyes of consumers, it is important that they are a vibrant shade of yellow. The anticipated result will therefore be achieved with low a* and high b* and L* values from the dried product's color characteristics. Higher b* and L* readings and reduced redness were the intended outcomes of the [40°C + 90 W] system and the [50°C + 90 W] system, respectively. In the process of drying fruits by microwave and hot air drying techniques, the change in their color is of high importance, and it is desired to minimize the change compared to the original color (Mana et al. 2012). The computed ∆*E* values indicated that the initial fruit color was better preserved by the [50°C + 90 W] procedure. It might be argued that the length of temperature exposure was efficient in producing this result, despite the higher temperature. Maskan (2001) examined the color changes and kinetics in kiwi slices when dried with hot air (60°C), microwave (210 W), and [hot air + microwave] systems. He stated that the effect of hot air drying on color parameters was more limited than other drying methods and that there were undesirable changes in color with the introduction of microwave. Mana et al. (2012), who dried okra with microwave (500, 600, and 800 W) and hot air (40°C and 50°C), pointed out the microwave drying as the optimum method that provides the minimum effect on total color change and offers the least drying time. According to Li et al. (2018), when using several drying techniques on apple cubes, the hot air drying system at 40°C produced unfavorable outcomes in terms of color alterations. Khodifad and Dhamsaniya (2020) reviewed microwave as the method that provides the least discoloration in fruits with various studies.

Crispness as one of the key factors in influencing consumers’ decisions when it comes to dried snacks is commonly measured using HA (Li et al. 2018). However, when the crispness on the surface of the product gives way to a soft, easy‐to‐chew, and nonsticky inner texture, the flavor can be delightful for consumers. Therefore, the versatile analysis of the textural properties of snacks is crucial. The HA characteristic of the apple cubes exhibited an upward trend with the microwaving incorporation, but the 120 W level caused a statistically insignificant drop, according to an evaluation of the measured results in Table 2. Hardening may have intensified due to the cubes’ quick drying and abrupt water loss with microwave. In terms of elasticity (SP) parameter, it can be said that the drying treatments did not make a marked difference on the products, except for [50°C + 90 W] process. CO values generally decreased with microwave involvement in all drying applications, although not significantly in 50°C treatments. The GU values of the products increased numerically as the microwave energy was involved. Nevertheless, the microwave energy increasing from 90 to 120 W had a decreasing effect on the GU of the apple cubes. Given that CO and HA are the sources of the GU parameter in technical measurement, this mechanism of action is similar to that of HA. In both drying techniques, the apple cubes’ CH properties, derived from GU and CO, were found to be higher. In addition, the CH change appears to be more significant in drying procedures of 50°C. In comparison to the 40°C operations, there was comparatively less RE, or the capacity to regain its original shape following compression, in the 50°C applications. It was determined that the RE values decreased with the introduction of microwave. Süfer et al. (2018) revealed the supportive results by examining the texture profile of onion slices dried by convective and microwave methods. They found that the parameters of HA, CH, SP, and GU were higher in the microwave application than in the convective method. On the other hand, they indicated that when the temperature and microwave power rose, the measurements for CH and HA improved.

All these changes in the texture profile of the dried apple cubes were initiated by ultrasonication and followed by osmotic dehydration and probiotic infusion. Clearly, due to the diffusion of water and other substances between the fruit and the solutions, the porous structure and water content of the fruit changed, and the main drying applications were directly affected in terms of drying time. In particular, the change in drying time, the amount of water in the final product, and the application of microwaves are interpreted as the most important factors in the formation of the final products’ texture characteristics. The review studies of Durance and Yaghmaee (2011) and Khodifad and Dhamsaniya (2020), which include a range of investigations, corroborate this interpretation and conclusion.

**FIGURE 2:**
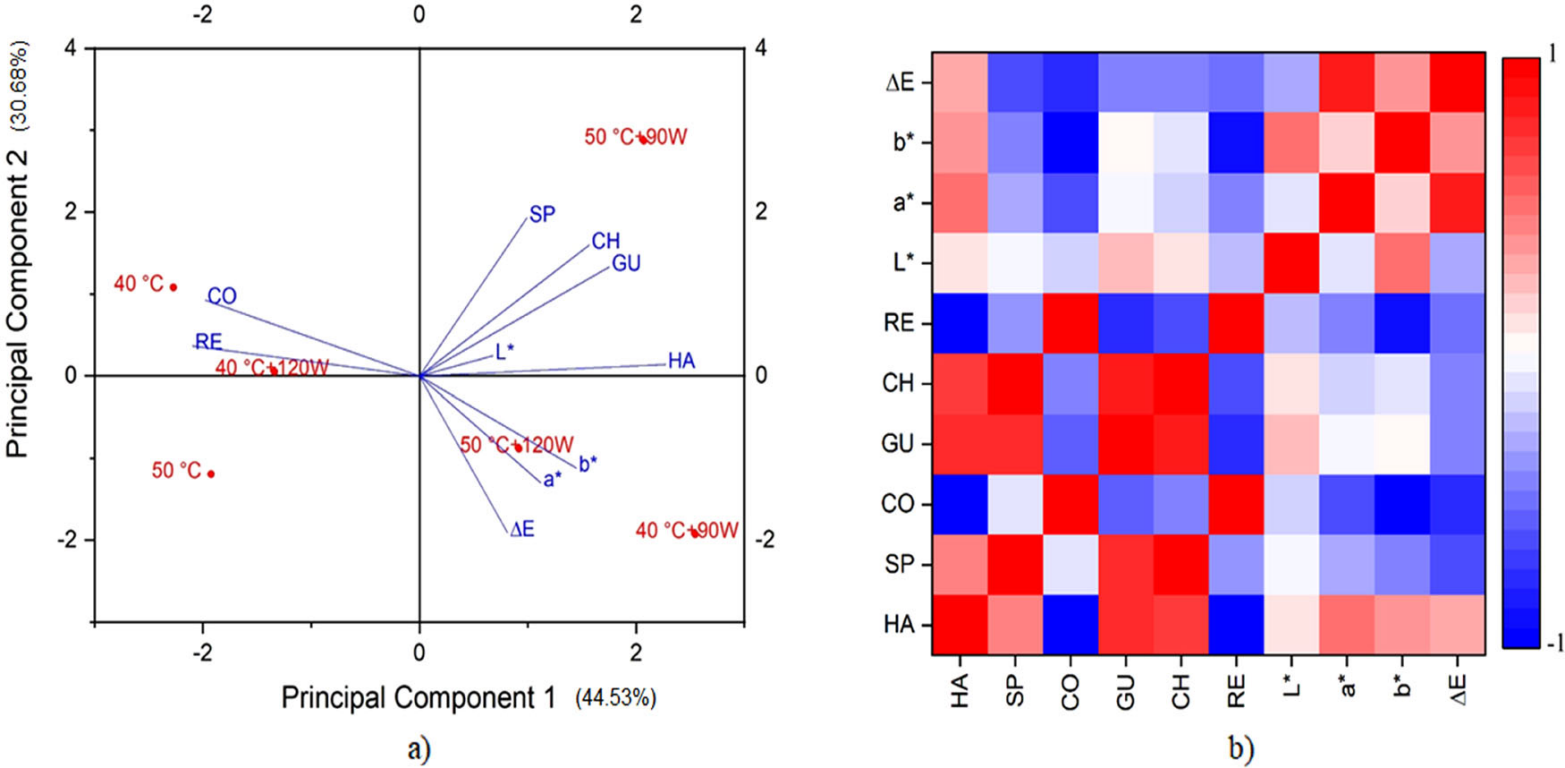
Multivariate representation of the dried apple cubes: (a) biplot of principal component analysis and (b) Pearson correlation analysis matrix, heat map. CH, chewiness; CO, cohesiveness; GU, gumminess; HA, hardness; RE, resilience; SP, springiness.

The application of PCA was employed to better understand the interactions between the dependent variables of color (L*, a*, b*, ∆*E*) and texture (HA, SP, CO, GU, CH, RE) of the apple cubes dried by two operations. Kaiser's criteria (Kaiser 1960) were followed in evaluating the model, which produced two major principal components and a total explained variance percentage of over 70%. The second principal component described 30.68% of the variability, whereas the first principal component accounted for 44.53%. The biplot graphic (Figure 2a) illustrates how PCA analysis can depict the connections between the various quality parameters of the dried apple cubes. A very strong association between a*, b*, and ∆*E* was revealed by the PCA results. A positive correlation was found in Principal Component 1 (PC1) and a negative correlation in Principal Component 2 (PC2) among the parameters a*, b*, and ∆*E*. This indicates that, as PC1 is dominant, flexibility increases or decreases with increasing priority. The PCA results revealed a very strong relationship among L*, SP, CH, GU, and HA, with a positive correlation on both PC1 and PC2. The relatively increasing role of microwave energy on these parameters individually may cause this positive correlation. The texture features CO and RE show a negative correlation for PC1 and a positive correlation for PC2; however, it can be stated that there is a strong positive correlation between them. In other words, as one increases or decreases, the other tends to increase or decrease as well.

There are various degrees of correlation between the different quality parameters investigated with Pearson correlation analysis (Figure 2b). The parameter of Δ*E* was found to correlate strongly with a* and b* parameters, and the HA parameter was also observed to have a relationship with Δ*E*, a*, and b*. The parameters of HA, SP, GU, and CH all showed a direct and strong relationship with each other. CO and RE were found to strongly correlate with each other. It is possible that the effects of rising temperatures and microwave involution led to these correlation findings.

### 3.3 Evaluation of Water Removing and Energy Consumed

**FIGURE 3:**
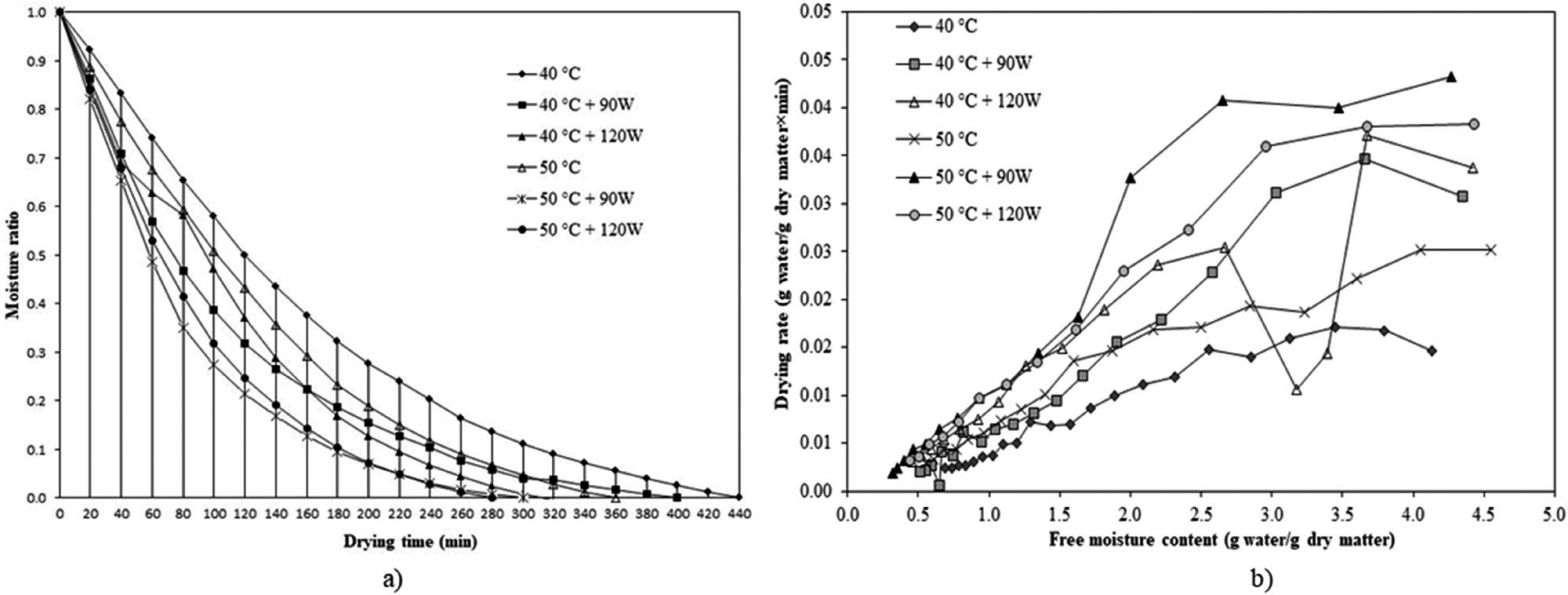
Curves of the moisture ratio (a) and the drying rate (b) at different process conditions.

Figure 3a illustrates the effects of MR and time on the drying processes under various conditions. The drying time increases as both temperature and power decrease. Under the same temperature conditions, it is observed that the drying time decreases as the applied power increases. In addition, at constant power levels, an increase in temperature further reduces the drying time. This indicates that both higher temperature and increased power facilitate the accelerated removal of moisture. Therefore, as the temperature increased, the drying rate increased, and the drying time decreased. Similar results were reported by Seiiedlou et al. (2010), Darvishi et al. (2013), Zarein et al. (2015), and Beigi (2016). The shortest drying time for the apple cubes was recorded as 280 min at [50°C + 120 W], whereas the longest drying time was recorded as 440 min at 40°C.

It is known that fruits and vegetables mostly dry in the falling rate period (Süfer et al. 2018; Wang et al. 2019; Zhou et al. 2019). The falling drying rate periods of the apple cubes are shown in Figure 3b. The drying condition at [40°C + 120 W] exhibited a different drying curve compared to other drying conditions. This difference is attributed to the combination of low temperature and high power, which results in a slower drying rate than in other processes and causes it to enter the falling rate phase earlier or in a different manner (Zarein et al. 2015; Horuz et al. 2020). It has been reported that the use of very high temperature and microwave power results in an initially much higher drying rate compared to other methods. Consequently, moisture decreases rapidly, leading to an earlier entry into the falling drying rate stage or at a different rate (Arslan and Özcan 2011).

Fick's diffusion model (Fick's second law) was employed to analyze the moisture transfer behavior during the drying of the apple cubes. Effective moisture diffusivity was calculated using the slope method and the diffusion model equation. According to Olanipekun et al. (2014), moisture diffusivity values for food products typically fall within the range of 10^−11^–10^−6^ m^2^/s. In our study, the moisture diffusivity values of apple cubes under different drying conditions were found to range between 2.68 × 10^−9^ and 1.55 × 10^−9^ m^2^/s (Table 3). In the study of Nuñez et al. (2024), probiotic‐enriched apple slices (*Lacticaseibacillus rhamnosus*) were dried using refractive windows and traditional hot drying, with *D*_eff_ values of 1.22 × 10^−9^ and 0.87 × 10^−9^ m^2^/s, respectively. These findings are comparable to the *D*_eff_ values obtained in our study. İzli and Polat (2018) observed that *D*_eff_ values for intermittently microwave‐dried apple slices ranged from 8.11 × 10^−9^ to 1.22 × 10^−8^ m^2^/s, whereas Tepe and Tepe (2020) reported that *D*_eff_ values were between 4.47 × 10^−9^ and 2.54 × 10^−8^ m^2^/s for intermittent microwave drying and between 3.38 × 10^−10^ and 6.25 × 10^−10^ m^2^/s for hot air drying. These results are consistent with those obtained by Beigi (2016) for conventionally dried apple slices, with *D*_eff_ values ranging from 7.03 × 10^−10^ to 1.08 × 10^−9^ m^2^/s. As reported by Zarein et al. (2015), the effective diffusion coefficient increases with rising microwave power due to the increased heating energy, which enhances activities of water molecules. Similarly, in our investigation, *D*_eff_ increased with both temperature and microwave power. However, a comparison between the [50°C + 120 W] system and the [50°C + 90 W] system revealed a decrease in the *D*_eff_ value. These variations could be attributed to differences in initial moisture content, drying temperature, equipment, as well as the composition and physical structure of the food (Doymaz 2017).

**TABLE 3:** The values of Biot number, effective moisture diffusivity, consumed energy in the dryer, and calculated specific energy consumption.

Drying conditions	*B_i_*	*D*_eff_‐Fick × 10^9^ (m^2^/s)	^a^Energy consumed (kWh)	SEC (kWh/kg)
40°C	0.4525	1.55	0.92	13.10
40°C + 90 W	0.7425	1.96	1.11	15.88
40°C + 120 W	0.5676	2.35	0.96	14.41
50°C	0.4940	1.93	0.90	11.84
50°C + 90 W	0.7124	2.68	0.98	14.06
50°C + 120 W	0.6528	2.61	0.92	13.65

Abbreviation: SEC, specific energy consumption.

^a^Energy consumption measured in the dryer.

SEC plays a crucial role in the development of energy‐efficient drying technologies for food materials. The SEC of the apple cubes ranging between 11.84 and 15.88 kWh/kg across different drying conditions is shown in Table 3. The lower energy consumption observed in some cases can be attributed to the use of higher temperatures and increased microwave power, which accelerate the drying process and reduce overall energy usage (Demirel and İsmail 2017; Çelen 2019). As shown in Table 3, energy consumption decreased as the drying temperature increased. Additionally, it was determined that energy consumption also decreased with increased microwave power at the same temperature.

The Biot number (*B_i_*) indicating resistance to moisture diffusion within the samples is a critical dimensionless parameter in the drying process (Dincer and Hussain 2002). The *B_i_* values ranged from 0.4525 to 0.7425 which are within the typical range for drying processes (0.1 < *B_i_* < 100), indicating comparable external and internal resistances. This suggests that the drying process is primarily controlled by external mass transfer (Górnicki et al. 2019).

In terms of the amount of energy used in drying, it can be observed that there is not much difference between the processes. Overall energy usage fell slightly as a result of the higher drying temperature and shorter drying time. The [50°C + 120 W] drying system would be the best option if we had to choose one that would use the least amount of energy, achieve the lowest amount of moisture content, and need the shortest drying time. But, this system with 0.10 × 10^5^ CFU/g result does not provide the intended outcome, as the major goal of this study is to attain the highest number of viable cells possible. The drying process with the lowest temperature, 40°C, naturally resulted in the maximum viable cell count of 2.28 × 10^7^ CFU/g. On the other hand, considering the final moisture content value of this process, it is clear that it is unreasonable in terms of potential risks such as unwanted microorganism growth and excessive soft tissue that could arise in the finished product. In the present situation, it would make more sense to assess the quantity of viable cells along with the fundamental quality parameters in the finished product. In this case, the [50°C + 90 W] drying condition could be the optimum system to be applied for the production of probiotic‐infused apple cubes based on an assessment of all the data.

### 3.4 Evaluation of Drying Kinetics

Table 4 presents the statistical values of the six drying models and the ANN. Lower RMSE and higher *R* ^2^ values indicate better model fitting (Okonkwo et al. 2022). The table shows that all applied models achieved high *R* ^2^ values (between 0.9783 and 0.9999) and low RMSE values (ranging from 0.0021 to 0.0475), indicating good model fits. The Midilli and others model provided the best fit under the drying conditions of [40°C + 90 W] and [50°C + 120 W]. İzli and Polat (2018), Beigi (2016), Zarein et al. (2015), Sharabiani et al. (2021), and Tepe (2024) similarly reported that the Midilli and others model gave best fit for the MR of microwave‐dried apple slices. In contrast, the ANN outperformed in the other drying conditions. Tepe (2024) performed mathematical modeling of apple slices using ANNs in MATLAB using three inputs and moisture content as output parameters and found *R* ^2^ values as 0.9999 for all experimental conditions. Additionally, Jafari et al. (2016), Karakaplan et al. (2019), Ghasemkhani et al. (2021), Sharabiani et al. (2021), and Tepe (2024) reported that ANN performs better than thin‐layer models for MR prediction.

**TABLE 4:** The results of the modeling approaches and statistical evaluations.

Models	Parameters	Drying conditions					
		40°C	40°C + 90 W	40°C + 120 W	50°C	50°C + 90 W	50°C + 120 W
Page	*R* ^2^	0.9988	0.9989	0.9902	0.9975	0.9988	0.9993
	RMSE	0.0110	0.0102	0.0325	0.0163	0.0111	0.0090
	*k*	0.0016	0.0069	0.0028	0.0021	0.0070	0.0049
	*n*	1.2623	1.0647	1.2424	1.2663	1.1296	1.1867
Newton	*R* ^2^	0.9831	0.9977	0.9976	0.9820	0.9951	0.9916
	RMSE	0.0407	0.0140	0.0475	0.0425	0.0218	0.0293
	*k*	0.0064	0.0095	0.0091	0.0078	0.0126	0.0116
Wang and Singh	*R* ^2^	0.9984	0.9721	0.9951	0.9992	0.9783	0.9934
	RMSE	0.0127	0.0506	0.0230	0.0095	0.0474	0.0270
	*a*	−0.0047	−0.0064	−0.0065	−0.0057	−0.0086	−0.0083
	*b*	5.7 × 10^−6^	1 × 10^−6^	1.1 × 10^−6^	8.3 × 10^−6^	1.8 × 10^−6^	1.7 × 10^−6^
Logarithmic	*R* ^2^	0.9987	0.9992	0.9947	0.9986	0.9980	0.9990
	RMSE	0.0118	0.0089	0.0246	0.0125	0.0150	0.0111
	*a*	1.1622	1.0346	1.1678	1.1722	1.0526	1.0925
	*c*	−0.1309	−0.0219	−0.1694	−0.1537	−0.0300	−0.0718
	*K*	0.0051	0.0091	0.0065	0.0059	0.0119	0.0101
Midilli and others	*R* ^2^	0.9999	0.9991	0.9949	0.9996	0.9989	0.9999
	RMSE	0.0021	0.0088	0.0253	0.0070	0.0117	0.0040
	*a*	1.0008	1.0103	0.9805	0.9916	1.0061	1.0015
	*n*	1.1794	1.0164	1.1270	1.1748	1.0061	1.1278
	*b*	−0.0010	−0.0004	−0.0002	−0.0002	−0.0002	−0.0009
	*k*	0.0023	0.0086	0.0042	0.0029	0.0077	0.0061
Henderson–Pabis	*R* ^2^	0.9888	0.9983	0.9797	0.9862	0.9964	0.9938
	RMSE	0.0339	0.0124	0.0468	0.0383	0.0193	0.0261
	*a*	1.0705	1.0230	1.0411	1.0595	1.0345	1.0436
	*k*	0.0069	0.0097	0.0094	0.0083	0.0130	0.0120
ANN	*R* ^2^	0.9999	0.9961	0.9997	0.9999	0.9996	0.9995
	RMSE	0.0245	0.0245	0.0155	0.0066	0.0157	0.0184

Abbreviations: ANN, artificial neural network; RMSE, root mean square error.

**FIGURE 4:**
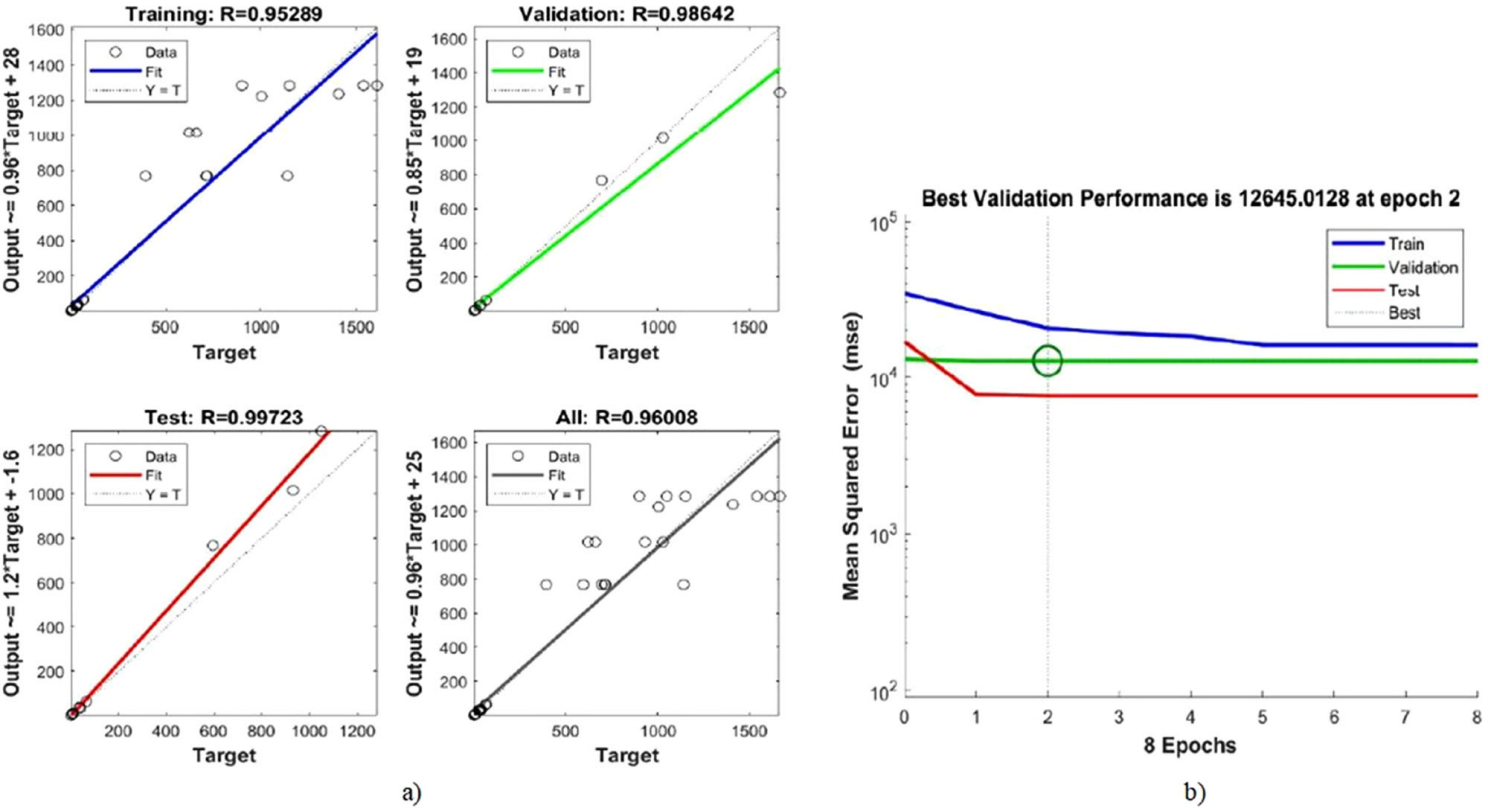
ANN modeling results: (a) regressions of ANN modeling and (b) best validation performance of ANN.

In comparison to the mathematical models, the ANN method provides deeper insight into the modeling and prediction processes by illustrating the relationships between input and output components through a set of rules that govern data modification. Figure 4 presents the regression plots and mean squared error (MSE) values of the model constructed using a multilayer feed‐forward architecture to assess the quality parameters of the ANN. The results closely align with the expected trend line, highlighting the model's predictive accuracy on the test data. These findings underscore the ANN model's ability to reliably predict test data and effectively capture the complex relationships between input and output variables. The model's robustness is further indicated by a high regression coefficient of 0.96008. El‐Mesery et al. (2024) modeled ANNs to predict garlic quality during hybrid infrared‐convective drying. In this study, a multilayer feed‐forward ANN model was developed in MATLAB, incorporating speed, temperature, and radiation as input parameters, while drying time, rehydration rate, allicin content, color characteristics, flavor profiles, and effective moisture diffusivity (*D*_eff_) served as output parameters. The model achieved regression coefficients ranging from 0.97415 to 0.99633, indicating high accuracy across various conditions. The results of this study align closely with those of our research, further supporting the predictive accuracy and reliability of the model.

## 4 Conclusions

It is clear that more water is extracted and energy is conserved at the maximum temperature (50°C) and microwave power (120 W). However, the drying operation at the highest temperature but lower microwave power (90 W) appears to be able to provide the desired probiotic‐infused apple cubes in terms of a relatively high viable cell, a moderate drying time, and parameters representing the color and texture elements that the viability is visually influenced by at the time of purchase. It is obvious that the model developed by Midilli and others makes the most accurate predictions for all operations. The study has been enhanced by the examination of multiple texture parameters and how they relate to color parameters. The predominant relationship of HA, both alone and with GU and CH, as well as with color parameters, is noticeable. Even in systems with multiple applications, such as US‐OD, probiotic microorganism impregnation, and drying under different conditions, modeling the drying process with ANN was able to provide high accuracy prediction.

There could be several recommendations for further analysis to eliminate various limitations in the study and to provide a more detailed approach: Changes in texture and color can also be observed before drying processes. In particular, it may be meaningful to analyze whether the texture parameters are altered by US‐OD treatment. The possibility of penetration of microorganisms by the sugar solution and ultrasonication can be measured. After the three main treatments applied to the apple cubes—US‐OD, probiotic bacteria infusion, and drying—SEM analysis can be used to investigate the change in the porous structure of the cubes and how it affects the next step.

## Nomenclature

*R* ^2^
: coefficient of determination
∆*E*
: color change
W: microwave power
*M*
: moisture content at any given time
*M*_0_
: product's initial moisture content
*M_e_*
: product's moisture content at equilibrium
*M_(t_*_+Δ_ *_t_*_)_
: moisture content in dry material at any time
∆*t*
: drying time range
*t*
: drying time
*D*_eff_‐Fick: Fick's second law of diffusion
*D*_eff_ *_,_*
effective diffusivity coefficient
*n*
: number of terms of the series
*L*
: average value of the side length of apple cube
*B_i_*
: Biot number
*D_i_*
: Dincer number
*v*
: air velocity
*L*
: thickness of apple cube
*k*
: drying constant
*E_t_*
: data measured by a watt meter
*M_w_*
: initial weight of fresh sample
*N*
: number of data
MR_pre_ *_,i_*
: predicted model value of moisture ratio
MR_exp_ *_,i_*
: experimental value of moisture ratio
*P*
: probability value of statistical analysis

## Author Contributions

**Derya Dursun Saydam**: conceptualization, investigation, funding acquisition, writing–original draft, methodology, validation, visualization, writing–review and editing, software, formal analysis, project administration, data curation, supervision, resources.

## Conflicts of Interest

The author declares no conflicts of interest.
